# Are parents aware of their children's hearing complaints?

**DOI:** 10.5935/1808-8694.20120005

**Published:** 2015-11-20

**Authors:** Keila Alessandra Baraldi Knobel, Maria Cecília Marconi Pinheiro Lima

**Affiliations:** a PhD; Researcher; Post-Doctoral Student - School of Medical Sciences - State University of Campinas; b PhD; Assistant Professor - Graduate Program in Speech and Hearing Therapy - Medical School of the State University of Campinas

**Keywords:** child, hearing, hyperacusis, parents, tinnitus

## Abstract

The accuracy of parents' impressions about their child's hearing status is variable and may not correspond to the child's complaints.

**Aim:**

To investigate children's self-reported hearing symptoms and parents' impressions about it.

**Methods:**

477 children (2^nd^ to 5^th^ grades of elementary schools) were interviewed and parents answered a survey at home. There were 393 matches between the children's interview and the parent's survey.

**Results:**

29% of the children reported trouble in understanding what people said, 36.1% had history of 1-3 ear infections, 12.7% had four or more ear infections, 21.7% had continuous tinnitus (positive association with history of exposure to loud sounds, *p =* 0.0007), 3.8% had pulsatile tinnitus and 2.9% had auditory hallucinations. 28.5% of the children were annoyed by loud sounds (associated with tinnitus, *p* = 0.0142, and gender, *p =* 0.0029) 10.4% had had audiological tests, and the determinant factors were history of ear infections (*p* < 0.001) and parents' concern about their child's hearing (*p* = 0.043). Parents and their own child's responses were significantly different.

**Conclusions:**

Children's auditory complaints were prevalent and relevant, but most of them had never had an audiological evaluation and most parents were not aware of their child's complaints. Sound intolerances and auditory hallucinations should be considered in clinical and audiological examinations.

## INTRODUCTION

Communication occurs in many ways, but oral communication is the main mode of the educational system. For this reason, early identification of any type or level of hearing loss gives students the opportunity of an early intervention to prevent negative effects of the hearing loss on the child's speech, language, learning, and academic outcomes^1^^,^^2^.

The prevalence of hearing impairment in children depends on how rigorous the criteria are to define it^1^^,^^3^^,^^4^ and on living conditions and access to health care^2^^,^^5^. According to the World Health Organization^3^, the prevalence of hearing impairment among school aged children ranges from 0.05% to 7.7%, but more recent studies found prevalence of 14%^6^, 14.9%^7^, 18.4%^8^, 19.5%^9^ and 24%^10^. In summary, a considerable number of children face hearing difficulties and need to be identified and receive proper assistance. In countries where hearing screening for school-aged children is not a routine, children can only count on adults' attention. However, studies have shown that parental concern for their child's hearing has low sensitivity and very low positive predictive values for detecting hearing loss, especially minimal or mild ones^4^^,^^11^^,^^12^.

Another auditory symptom that is common among children is tinnitus^13^. Tinnitus is a phantom auditory perception, since it is a perception that is not related to an external source^14^, ^15^, ^16^. The prevalence of tinnitus among children varies from 6% to 59%^6^. Despite the disclosed prevalence data, attributed to important differences in methods of data collection, diagnostic criteria, and age groups^6^, there is a consensus that it is higher among children with otological disorders or history of exposures to loud sounds^4^^,^^6^^,^^17^, ^18^, ^19^, ^20^. Children seem to be less distressed by tinnitus than adults, but children who complain of tinnitus should be taken seriously, since it may be a sign of an otological condition and can affect children's lives as it is reported to have on adults^18^.

Auditory hallucination (AH) is a different kind of phantom auditory perception. When the perception has the same qualities as a real one, meaning that the person does not recognize it as a distorted perception, it is called auditory hallucination (AH)^21^. The prevalence of AH in population based studies with adolescents from the general population is around 6%^22^^,^^23^, although the number of parents who reported hallucinations in their children was less than 1%^22^. In a follow-up research, Dhossche et al.^22^ reported that, eight years after they found 6% of adolescents (out of 914) from the general population with self-reported hallucination, no subject was diagnosed with schizophrenia. However, AH were associated with non-psychotic psychiatric problems, such as specific phobias, depressive disorders, substance use disorders, post-traumatic stress disorder and social phobia. Similar development was found in 16 (out of 90) non-psychotic children (5-12 years old) that reported AH in psychiatric clinics^24^.

Hyperacusis is defined as a decreased sound tolerance to ordinary sounds, even in low intensities^25^. Annoyance or feeling of displeasure with specific sounds can be called phonophobia (fear of sound) or misophonia (dislike of sound)^26^. There is scarce published information on the prevalence, possible associated causes and prognosis of decreased sound tolerance in childhood. A link between hyperacusis and tinnitus has been described in adults^25^, ^26^, ^27^ and in children^28^. According to Coelho et al.^28^ 50% of the children who had hyperacusis also reported tinnitus, and of those who did not have hyperacusis, 17.8% reported tinnitus.

In summary, population studies show that a significant number of children have at least one hearing complaint that can potentially cause negative effects on the child's communication, academic outcomes and emotional well-being. The aims of the present study were to evaluate the parents' impressions about their child's hearing, the prevalence of self-reported hearing impairment, tinnitus, AHs and decreased sound tolerance among children attending the 2^nd^ to 5^th^ grades of elementary school and to investigate possible risk factors for the complaints. The present study is part of a larger one that also sought information about children's knowledge, habits, preferences and protective behaviors regarding loud sound exposures^29^.

## METHOD

### Design

A prospective cross-sectional study was carried out in elementary schools in Campinas, a southeastern Brazilian town with 1.1 million inhabitants. Data were collected between April 2010 and November 2010.

The survey started after a pilot test with 60 children and their parents. We selected the questions of questionnaire with the children and the questions of the survey to the parents that were most suitable for the research.

### Selection criteria

In Campinas there are 72,326 students from 2^nd^ to 5^th^ grades^30^. Sixty per cent of them are distributed in 98 State Public Schools, 25% in 42 Municipal Schools and 15% in approximately 180 Private Schools. It is important to explain that in Brazil there are free municipal and state public schools and private schools. With few exceptions, public schools have limited educational resources and, for this reason, families with higher incomes usually send their children to private school. Children go to school either during the morning or afternoon. Children from 2^nd^ to 5^th^ grades are 6 to 10 years-old.

The city is divided into five regions. The selection of the schools was made in alphabetical order for each region. After a telephone contact, many school directors refused to receive us. So, the next school in alphabetical order in the same region was contacted. Using these criteria we selected 13 schools: seven State public, three Municipal public schools and three private. In large schools, with more than one class per year, the class was chosen also by letter (2^nd^, 3^rd^, 4^th^ and 5^th^ years C, for example). In small schools students either from the morning or from the afternoon period were interviewed.

With these procedures we had a selected sample of 753 children. Children with medical records of mental or psychiatric disabilities were excluded. Parents (mother, father or caretaker) received the informed consent form and a survey (described in assessments) to be filled out at home. Teachers collected the returned forms and questionnaires and gave them to the researcher, who went to the school to make the assessments. The number of interviewed children was 477. Considering the number of the selected sample and the number of interviewed children, the drop-out range was 36.8%. Withdrawals were due to no returns of the informed consent form (71.4%), parental refusals (23.2%), and children's non attendances at school at the day of the interview (5.4%). Because some parents' surveys were returned in blank, there were 393 matches between child's interview and his/her own parent's survey.

### Procedure

The interviews with the children were conducted during school-time. All children gave verbal consent and were individually and privately interviewed by the first author in the most silent room available in the school. Care was taken to ensure that children understood the questions and had plenty time to respond. The interviewer did not express either approval or disapproval to the child's answer at any time.

### Assessments

The parents' survey sought information on parental impressions of their child's auditory behavior and complaints, history of exposures to loud sounds, the number of episodes of otitis media and background information (8 items, Appendix A). The children's interview consisted of an open-ended questionnaire, with guideline questions presented orally by the interviewer about demographic information, self-reported trouble hearing in quiet, phantom auditory perceptions, sound annoyance and information about previous hearing tests (5 items, see Appendix B). Many children and adults described their tinnitus as sounds of insects, such as a bee or mosquito, tones and noises. But, to be considered tinnitus, these phantom auditory perceptions must be recognized by the person as “sounds” produced by their own ears or head. On the contrary, if the person does not recognize it as a distorted perception, it is called AHs^21^. For this reason, children that reported they hear insects, tones or noises around them, but could identify where it came from, believing it to come from somewhere outside themselves, were considered to have AHs. We had not planned a question about the level of annoyance with phantom auditory perceptions, but a discussion of partial results showed its importance. For this reason, only a sub-group of the participants (239 children from state public schools) were answered to this question.

### Statistical analysis

There was a content analysis for the questions that included descriptions. Those responses were coded prior to statistical analysis. Statistical analysis was performed by using SAS (version 9.1) and the significance level was 5%.

Homogeneity and other exposure factors were analyzed by using Chi-square Test for percentage differences and Fischer's Exact Test for absolute frequencies (n < 5). Analysis of agreement in measurement comparing children's and parents' questionnaires was accomplished by using the Symmetric Test. It analyses how different the answers were.

### Ethics

The present study was approved by the ethics committee of research (number 940/2009). Written consent was obtained from all parents and verbal consent from all children.

## RESULTS

Samples from public and private schools were similar in gender and age, but Chi-Square Test showed a significant difference among schools with regard to parents' educational level (Table 1).

**Table 1:** Age, gender and parents' educational level according to public and private schools.

	Municipal public school			State public school			Private school			Total			*p*-value
	n		%	n	%	n		%	n		%		
Age	146		30.6	239		50.1	92		19.3	477		100	0.0636
Mean (years old)		8.5			8.2			8.2			8.3		
Standard-Deviation		1.3			1.1			1.2			1.2		
Gender													0.049
Female	77		52.7	105		56.1	34		37.0	216		45.3	
Male	69		47.3	134		43.9	58		63.0	261		54.7	
Total	146		100	239		100	92		100	477		100	
Parents' educational levels													< 0.0001
Missing data	21												
Elementary uncompleted	56		56	40		40	4		4	100		24.8	
Elementary completed	22		50	21		47.7	1		2.3	44		10.9	
High-School	55		34.2	99		61.5	7		4.3	161		40.0	
Bachelor and graduation	8		8.2	20		20.4	70		71.4	98		24.3	

Chi-SquareTest.

Twenty nine percent of the children reported some trouble understanding what people say, 0.6% said they always had some trouble. According to parents' information, 36.1% of the children had history of one to three ear infections, 12.7% had had four or more ear infections and 2.5% did not know. The number of previous ear infections was not associated with parent's educational level (*p* = 0.639, Chi Square Test).

Despite the prevalence of self-reported hearing complaints and history of ear infections, only 10.4% had had an audiometric test, and almost 2/3 of them were from private schools. Table 2 displays the association of the audiometric test taken with parent's educational level, children's and parents' complaints and history of ear infections.

**Table 2:** Audiometric test according to parent's educational level, children's and parents complaints and history of ear infections.

Audiometric Test							
	Yes		No		Total		*p*-value
	n	%	n	%	n	%	
Parents' educational level							0.2094*
No response	128						
Elementary uncompleted	5	6.1	77	93.9	82	100.0	
Elementary completed	4	11.4	31	88.6	35	100.0	
High school	14	9.5	133	90.5	147	100.0	
Bachelor or Graduation	15	15.2	81	84.4	96	100.0	
Child's hearing difficulty							1.000
No response	36						
No complaint	33	10.5	280	89.5	313	100.0	
Sometimes	13	10.4	112	89.6	125	100.0	
Always	0	0.0	3	100.0	3	100.0	
Parent's complaint about their child hearing							0.043
No response	126						
No complaint	24	8.7	252	91.3	276	100.0	
Sometimes	11	14.7	64	85.3	75	100.0	
Always	3	37.5	5	62.5	8	100.0	
Do not know	0	0.0	3	100.0	3	100.0	
Ear infections							<0.0001
No response	125						
None	11	6.2	166	93.8	177	100.0	
1 to 3	9	6.9	122	93.1	131	100.0	
4 or more	17	36.2	29	61.7	47	100.0	
Do not know	1	11.1	8	88.9	9	100.0	

Chi-Square Test

* Fisher's Exact Test.

Excluding missing data from 13 children, the number of children with phantom auditory perception was 135 (28.4%). After classifying the perceptions, three categories were found: continuous tinnitus, such as a continuous ring or hum (n = 103/21.7%), pulsatile tinnitus (n = 18/3.8%) and AHs (n = 14/2.9%).

Table 3 provides the number and gender of children and the descriptions of their AHs. Owing to the small number of subjects with AHs, parametric tests were not performed. History of previous ear infections and previous exposure to loud sounds, gender and parents' educational level were examined with a view to investigating their association with complaints of trouble hearing and tinnitus (continuous and pulsatile) (Table 4).

**Table 3:** Number of descripted auditory hallucinations, gender and level of annoyance.

			Level of annoyance		
			Not bothered at all	A little annoyed	Very annoyed
Insects	M/F	2	x		
Tones	F	1	x		
Noises	M	1	x		
Doorbell	F	1	x		
Paces	F	1		x	
A voice screaming	M	1		x	
Sounds of somebody at home moving things around and horns	M	1		x	
Voices Conversing Together	F	1		x	
Music And Cat	F	1		x	
Music and weird sounds	F	1			x
Own name been called by a voice	M/M	2			x
A voice giving orders	F	1			x

M: male, F: female.

**Table 4:** Association of continuous and pulsatile tinnitus and trouble hearing complaints with previous ear infections, previous exposure to loud sounds, gender and parents educational level.

	Tinnitus					Hearing trouble				
	Yes		No			Yes		No		
	n	%	n	%	*p*-value	n	%	n	%	*p*value
Ear infections					0.1505					0.3238*
No response	17		62			60		22		
None	58	29.4	139	70.6		135	68.5	62	31.5	
1 to 3	32	23.3	105	76.6		102	74.5	35	25.5	
4 or more	14	26.4	39	76.6		31	62.0	19	38.0	
Do not know	0	0	9	100		6	66.7	3	3.3	
Number of types of exposures to loud sounds					0.0007					0.0170*
0	11	11.6	84	88.4		62	80.5	15	19.5	
1	33	26.8	112	77.2		77	65.2	41	34.8	
2	44	31.7	95	68.3		87	63.5	50	36.5	
3 or more	33	34.4	63	65.6		108	75.5%	35	24.5%	
Gender					0.0576					0.0732
Female	64	29.6	152	70.4		143	42.8%	73	51.8%	
Male	57	22.0	202	78.0		191	57.2%	68	48.2%	
Parents' educational level					0.3056					0.2910
No response	21		63			59		25		
Elementary uncompleted	23	23.7	74	76.3		66	68	31	32	
Elementary completed	12	27.9	31	72.1		27	62.8	16	37.2	
High school	46	29.9	108	70.1		107	69.5	47	30.5	
Bachelor and graduation	19	19.6	78	80.4		75	77.3	22	22.7	

Chi-Square Test

* Fisher's Exact Test.

Regarding the question about sounds that bothered the child, 59.4% denied any annoyance with sounds, 28.5% were annoyed by loud sounds (noise or music), 2.9% high pitch sounds, 1.0% body sounds produced by others (snoring, cleaning accumulated mucus in the throat, swallowing), 3.5% scratching sounds (on iron, black-board, polystyrene…) and 4.7% referred to other sounds, such as a broom sweeping, a plastic cup being squeezed, a chair being dragged across the floor, sand-paper, etc.

The study of the associations of previous ear infections, previous exposure to loud sounds, tinnitus, gender and parents' educational level with sound annoyance with loud and high pitch sounds is shown in Table 5. Agreements among children's and their own parents' answers are shown in Table 6. Dashed cells refer to agreement among parent and child answers. A symmetric test analyses how different they were.

**Table 5:** Annoyance with loud and high pitch sounds according to studied variables.

	Annoyance with loud sounds					Annoyance with high pitch sounds				
	Yes		No			Yes		No		
	n	%	n	%		n	%	n	%	
Tinnitus (continuous/pulsate)					0.0142					0.2079*
No response	0		13			0		13		
Yes	46	38.0	75	62.0		6	5.0	115	95.0	
No	93	26.3	261	73.7		8	2.3	346	97.7	
Ear infections					0.4726					0.7502*
No response	30		53			1		82		
None	49	23.9	156	76.1		8	3.9	197	96.1	
1 to 3	41	29.3	99	70.7		3	2.1	137	97.9	
4 or more	17	33.3	34	66.7		2	3.9	49	96.1	
Do not know	2	22.2	7	77.8		0	0.0	9	100.0	
Gender					0.0029					0.3744
Female	78	35.1	144	64.9		8	3.6	214	96.4	
No	61	22.9	205	77.1		6	2.3	260	97.7	
Number of types of exposures to loud sounds					0.4063					0.4779*
0	23	24.0	73	76.0		3	3.1	93	96.9	
1	36	25.9	103	74.1		3	2.2	136	97.8	
2	38	30.2	88	69.8		2	1.6	124	98.4	
3 or more	42	33.1	85	66.9		6	4.7	121	95.3	
Firework exposure (child's answer)				0.4152					0.5187	
No response	0		12			0		12		
Yes	55	31.4	120	68.6		4	2.3	171	97.7	
No	84	27.9	217	72.1		10	3.3	291	96.7	
Parents' educational level					0.0414					0.4456*
No response	32		55			1		86		
Elementary uncompleted	24	24.0	76	76.0		2	2.0	98	98.0	
Elementary completed	6	14.0	37	86.0		0	0.0	43	100.0	
High school	54	33.5	107	66.5		6	3.7	155	96.3	
Bachelor and graduation	23	23.7	74	76.3		5	5.2	92	94.8	

Chi-Square Test

* Fisher's Exact Test.

**Table 6:** Agreements between children's and parents' answers.

Child considers his/herself to have trouble hearing	Parent consider child to have trouble hearing						
	No	Sometimes	Always	Do not know	Total	Agreement	*p*-value
No	230	40	2	2	271		
Sometimes	65	45	5	1	116		
						70.2%	0.0140
Always	3	0	3	0	3		
Do not know	2	0	0	0	0		
Total	297	85	10	3	395		
	Parent says the child complained about tinnitus						
Child says he/she has tinnitus							
		Yes	No	Do not know	Total	Agreement	*p*-value
Yes		25	73	2	100		
No		26	247	15	288	70.1%	<0.0001
Do not know		0	0	0	0		
Total	51		320	17	388		
	Parent says the child covers the ears when exposed to loud sounds						
Child says he/she is annoyed by loud sounds							
	Yes	Sometimes	No	Do not know	Total	Agreement	*p*value
Yes	28	34	27	3	92		
Sometimes	0	0	0	0	0		
						68.2%	<0.0001
No	52	76	159	24	311		
Do not know	0	0	0	0	0		
Total	80	110	186	27	403		

Symmetric Test.

## DISCUSSION

The present study investigated self-reported hearing complaints among Brazilian children attending the 2^nd^, 3^rd^, 4^th^ and 5^th^ grades of elementary schools and parents' perceptions about this.

In Brazil, students do not pay to go to public schools. Family incomes were not analyzed, but the significant difference of parents' educational levels between municipal public, state public and private schools (*p* < 0.0001) seems to be influenced by other socioeconomic variables, such as access to adequate health care, housing, nutrition, etc. For this reason, we decided to analyze the variable parents' educational level across self-reported hearing complaints and not the school “type”. Children's age was similar among schools, but there were more boys than girls (*p* = 0.049), which correspond to the gender distribution in the city for this age range^30^.

### Hearing difficulties

Previous researches associated hearing difficulties to ear infections and lower incomes^2^^,^^31^ Although the majority of the Brazilian children who go to private schools are wealthier than the others, history of previous ear infections was not significantly different between public and private schools (*p* = 0.2000, Fisher's Exact Test). Contrary to other studies^2^^,^^32^, we did not find an association between children's hearing difficulties and history of ear infections, probably because of differences in methodology. In our study we investigated the self-reported hearing difficulty, not the hearing impairment, evaluated by both studies^2^^,^^32^ using puretone audiometry, tympanometry and otoscopy. Also, we gathered the total number of ear infections the child had in his/her life, while they looked for specific data, such as “otitis media treated with antibiotics”, “more than three episodes of otitis media in a year”^2^ and “otorrhea”^2^^,^^32^. Holgers & Pettersson^33^ found that the risk of subjective hearing loss increases with increasing noise exposure. Although there was an association between hearing difficulties and history of loud sound exposure in our study, it seems that there is a confounding variable affecting the hearing difficulty, since any number of exposures to loud sounds (from none to more than four) resulted in a higher percentage of children with complaints of hearing trouble.

Despite the auditory symptoms, only 10.4% of the children had ever had an audiometric test, with no statistical association with parents' educational level (*p* = 0.2094) or child's self-reported hearing complaint (*p* = 1.000). Only if the child was able to describe the hearing test was she considered as ever been evaluated. A previous hearing test was associated with history of ear infection (*p* < 0.0001) and parent's complaint about their child's hearing (*p* = 0.043). We did not interrogate parents about why they did or did not take their child for a hearing evaluation. Thus, we cannot say if parents took their child to have a hearing test because they were really worried about their child's auditory behavior or if they expressed their concern with their child's hearing because the child (maybe) failed in a hearing test that was ordered by a health care professional.

The symmetric test pointed to a significant difference between the parents and their own child's answers. In Table 5 we verify that, among the 116 children who had trouble hearing in silence, only 45 (38.8%) had parents that were aware of their difficulty. Though we found 29.6% of children with some hearing complaints (0.6% with more evident trouble) we cannot directly transpose it into actual hearing losses, since we did not evaluate their hearing and predictive values of parental suspicion of hearing impairment are yet to be discussed^4^^,^^34^, ^35^, ^36^. Despite those weak points, we totally agree with Cone et al.^4^ that a caregiver's concern with regards a child's hearing development is enough reason to prompt a formal hearing assessment.

### Tinnitus and AHs

Estimates of prevalence of tinnitus and decreased sound tolerance depend on the definitions adopted and on the way the questions are presented to the child. In order to have a better picture of those symptoms, we tried to identify all auditory phantom perceptions and sound annoyances and then we classified them. Tinnitus was reported by 28.4% of the children and was similar to the findings of Mills et al. (29%)^19^ and Raj-Koziak et al. (33%)^37^, but much higher than the findings of 9.2% from Holgers^17^ (12%) study and lower than Coelho et al. (37.5%)^6^ for the same age group.

We observed a significant association between tinnitus and the history of loud sound exposures^6^^,^^17^^,^^38^, which is worrying, since there is evidence that tinnitus may be an early warning sign of noise induced hearing loss^20^.

Only 51% of the parents reported that their child had tinnitus. So, despite the fact that half of the children did not talk to their parents about their tinnitus, we consider that our findings cast doubt on the assumption that it is rare for children to complain spontaneously of tinnitus to their parents^19^^,^^37^^,^^39^.

In our sample, 13 children (2.7%) reported auditory phantom perceptions that were compatible with AHs (Table 3). It is less than reported by other studies^22^, ^23^, ^24^, but it still shows that the symptom is not uncommon in childhood. Only one child had verbal hostile AHs, in which she told that she heard “her dead uncle giving orders to her”. Another child reported that she heard voices conversing together, but she said it was not possible to understand anything; it was more like a bubble noise. So, except for one child, the other AHs in this sample were predominantly nonthreatening^24^^,^^40^.

### Sound annoyance

We identified almost half of the children with some annoyance with sounds. Of course, our data is not enough to diagnose children with hyperacusis nor phonophobia, but our results are generally the same as those obtained by Coelho et al.^28^, who evaluated the Loudness Discomfort Level (LDL) of children within the same age group. Their study indicated that, among the children with LDL in the lowest 5^th^ percentile (lower than 90 dB HL), 42% were bothered by sounds, 3.2% had hyperacusis and 9% had phonophobia. Our study also supports that there is an association between tinnitus and annoyance with sounds (*p* = 0.0142) and that there is a higher occurrence of the complaint among girls (*p* = 0.0029), both found previously in studies with children^28^ and adults^41^.

### Limitations

One limitation of our study was the drop-out range of 36.8%, which decreases the epidemiological power of the study. Nevertheless, drop-out was similar in private and public schools and did not change the general age and gender distribution.

The attempt to find possible relations between hearing complaints and the number of types of noise exposures may have been limited because children self-reported noise exposures and we have no data about how frequent or infrequent those exposures were. It is also possible that some children did not remember taking an audiometric test.

### Future directions

Parental and teacher vigilance over children's hearing behavior and sensitive hearing screening programs are essential to identify children that should take a complete ear and hearing evaluation. Besides, we need to encourage children to talk about themselves or, at least, we have to start listening to their complaints and it follows that if the child has a hearing complaint, tinnitus, AHs or annoyance with sounds, something has to be done.

We also support the urgent need of hearing screening programs and pediatric ear health assistance for school-aged children. Ideally, hearing and audiological examinations should be incorporated into routine pediatric visits, especially for children of literacy age.

## CONCLUSION

Difficulties in understanding what people say, decreased sound tolerance, tinnitus and hearing hallucinations are common complaints among children. Despite the relevance of those auditory complaints, the majority of the parents were not aware of their child's complaint, and even when they were, most of the children did not take an audiological evaluation. Sound intolerances and AHs should be considered in clinical and audiological examinations.
